# Investigating causes of the high prevalence of sexually transmitted infections in Du Noon

**DOI:** 10.4102/safp.v66i1.5794

**Published:** 2024-03-19

**Authors:** Azhaar B.F. Dookhith, Adil Razack, Abdul-Aziez Isaacs

**Affiliations:** 1 Division of Family Medicine, Faculty of Health Sciences, University of Cape Town, South Africa; 2 Metro Health Services (Western Cape), Cape Town, South Africa

**Keywords:** sexually transmitted infections, primary health care, risk factors, knowledge, prevalence, HIV, health education, aetiology

## Abstract

**Background:**

In South Africa, sexually transmitted infections (STIs) represent a significant public health issue. Sexually transmitted infections contribute significantly to the burden of disease in South Africa and are recognised as one of the main causes of the human immunodeficiency virus (HIV) epidemic. The aim of this study was to investigate the potential causes of the high prevalence of STIs in the Du Noon population.

**Methods:**

A mixed methodology study involving 40 participants between the ages of 18 years and 45 years was conducted at Du Noon community health centre from 01 May 2021 to 15 May 2021. Both structured questionnaires and one-on-one patient interviews with open-ended questions were utilised to collect data.

**Results:**

Cultural beliefs, having multiple partners, a lack of partner notification, alcohol consumption, and a lack of condom usage were found to be the main contributing factors to the high incidence of STIs. Sex education appears to be lacking. Our findings reflected the other well-known cultural and socioeconomic issues confronting South African communities, for example, poverty, age-disparate relationships, and polygamous relationships.

**Conclusion:**

The cultural perspectives and understandings of sexual interactions of older men appear to have an impact on younger generations; as do peer pressure, social media and other socio-economic factors. There is an urgent need to shift cultural ideologies and norms among the youth. More research is needed to understand the views and misconceptions of the general public about STIs.

**Contribution:**

This study highlighted how health education challenges, interpersonal relationships, and socioeconomic barriers are still important factors in STI transmission.

## Introduction

Sexually transmitted infections (STIs) are a major public health concern in South Africa.^1^ South Africa’s burden of disease because of STIs is currently one of the largest in the world.^2,3^ The prevalence of STIs is relatively high and can lead to an increased risk of human immunodeficiency virus (HIV) transmission, adverse pregnancy outcomes, and infertility.^3,4^ The burden of HIV has historically been heavy and continues to be a serious public health problem in South Africa.^5^ Patients with STIs face an increased biological risk of HIV acquisition because of the virus’s invasion of the immune system through genital lesions and/or inflammation caused by STIs.^6^ In South Africa, numerous studies on STIs have been done in the last 10 years. Most of them have shown that there is a positive correlation between STIs and HIV. It is likely that concurrent STIs contribute to an increased HIV transmission rate.^3,4,7,8,9,10,11,12,13^

In South Africa, STIs continue to be a hidden epidemic; as of 2017, 7.9 million people were HIV-positive.^14^ In women between the ages of 15 years and 49 years, there were estimated to be 2.3 million new cases of gonorrhoea, 1.9 million new cases of chlamydia, and 23 175 new cases of syphilis; in men of the same age, there were estimated to be 2.2 million new cases of gonorrhoea, 3.9 million new cases of chlamydia, and 47 500 new cases of syphilis.^14^ Knowing the prevalence and modes of STI transmission is essential because of the connections between HIV and STIs.^15^

The epidemiology of STIs has received little attention in South Africa. Data collection from a fragmented healthcare system was made more challenging by the historical lack of interest in STIs as a health priority and the lack of a surveillance system. The illness burden has been estimated to some extent using facility-based ad hoc questionnaires. There is no comprehensive, well-rounded picture of the STI situation in South Africa.^15^ Other STIs are a significant contributing element to this type of transmission.^4,5,7,12,16^

A 2016 study showed a relatively high prevalence of bacterial STIs in South Africa, compared to other African countries.^12^ It is estimated that one in four women in South Africa is infected with at least one bacterial STI. The burden of STIs in South Africa is estimated to be higher in women than in men.^12,17^ A study done in 2020 on the high prevalence and incidence of STIs among women living in KwaZulu-Natal, South Africa, concluded that there is extremely high prevalence (83.3%) and incidence of (36.6%) STIs among HIV-positive women living in rural and urban communities in KwaZulu-Natal, South Africa.^4^

A descriptive cross-sectional study showed that knowledge of STIs did not appear to have any effect on perceptions of the risk of acquiring STIs or the relationship between STIs and HIV transmission.^8^ A study was done in 2017 in Malaysia among university students, assessing their knowledge, attitudes, risky behaviours, and preventative practices related to STIs. The conclusion was that knowledge on the non-HIV causes of STIs is still lacking, and the risky behaviour practised by the sexually-active students in this study was quite alarming.^18^

A study in 2019 concluded that the implementation of educational awareness programmes in schools and campaigns aimed at increasing knowledge about STIs in urban and rural areas at the national level will increase awareness and willingness for screening and will decrease STI transmission and discrimination in Africa.^19^

A study conducted in Eastern South Africa’s rural communities revealed that the main factor influencing the prevalence of HIV and other STIs is sexual behaviour.^7^ A different study found that there is a correlation between sexual conduct and age, and that younger women are more likely to have multiple partners at the same time and participate in riskier sexual behaviours. Women were less likely to know if their partners were HIV-positive, and those who were infected reported having had more sexual partners over their lives.^7^

A study in the United States (US) and other sub-Saharan African nations was conducted to address the issue of sexual behaviour and STIs. It was determined that there is a coherent variation in sexual behaviour among various ethnic groups and populations. One additional factor contributing to a greater risk of STIs is sexual activity.^20^ According to a 2007 study, women’s poor socioeconomic position has contributed significantly to the spread of STIs and continues to be a barrier to attempts to prevent STIs.^21^

The aim of this study was to investigate the potential causes of the high prevalence of STIs in the Du Noon population, with the objectives to gaining a better understanding of the knowledge and attitudes towards STIs among members of the Du Noon community, with a view to establish targeted interventions. Reducing the incidence of STIs can reduce high patient numbers, HIV transmission, adverse pregnancy events, and infertility. Specifically, measures will be taken to determine patients’ knowledge of STIs, attitudes towards sexual behaviour in culture and religion, the relationship between socioeconomic status and sexual behaviour, and the sexual behaviour of young adults.

## Research methods and design

### Study design

A mixed methodology study design was used, similar to that of Folasayo et al.^18^ This design has been chosen as it allows participants in the study to participate in an open-ended interview as well as complete a structured questionnaire in order to explore the reasons for the high incidence of STIs in this community.

### Setting

Du Noon is a small township situated in Milnerton, Cape Town, South Africa. Its population consists mainly of black African people (89.3%), who are mostly IsiXhosa speakers, with the rest being mixed race people (5.6%), white people (0.2%), Indian people (0.1%), and other people (4.9%).^22^ It has a formal and dedicated community health centre (CHC) that has been operational since 2015.^23^ Du Noon CHC has a large HIV-positive population of approximately 8000 people, and recent data from November 2019 to February 2020 showed that 1760 people are being treated for STIs.^22,23^

The study population consisted of young adults between the ages 18–40 years. This study was aimed at younger adults as older patients may be in the minority and have different reasons for re-infection than younger patients. Other inclusion criteria included a confirmed diagnosis of STI (based on the history and clinical examination), both males and females, and mentally competent to give informed consent. Pregnant patients were excluded, as their discharge may have been physiological and not because of an STI as well as patients not living in Du Noon.

**FIGURE 1:**
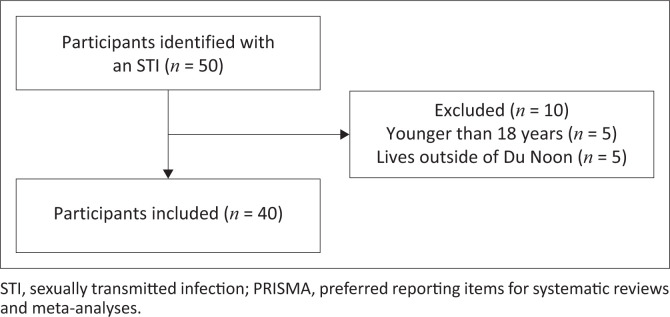
PRISMA flow chart indicating participants recruited.

A sample size of 171 was determined using an estimated 20% prevalence of STIs from the facility’s data, a 90% confidence interval, and a 5% margin of error.^24^ Although a random sample approach would have been ideal, it was not feasible because of constraints on time and resources, as well as the coronavirus disease 2019 (COVID-19) pandemic’s ban on patient admissions for less serious illnesses. Thereafter, the sampling strategy was purposive, and the final sample size was 40 participants (Figure 1).

During the COVID-19 pandemic, the Du Noon CHC attended to walk-in clients by screening for COVID-19 at the entrance of the clinic. Those without COVID-19 symptoms were allowed to form part of the walk-in queue. Strict precautions were taken by the health staff of the clinic by wearing personal protective equipment, applying social distancing among the walk-in patients, and using hand sanitisers. Consent was obtained both verbally and in writing prior to the interviews by the researcher.

### Data collection

Data were gathered using structured questionnaire-based one-on-one patient interviews. The utilised survey was modified from validated and reliable surveys from earlier research.^18,21,25^ The questionnaire builds on inquiries made in earlier research on the significance of partner notification, condom accessibility, and general knowledge of STIs.^18,21^ To increase the dependability of the responses, both positive and negative framing questions were employed. More specific information was obtained since each answer contained seven potential response elements. In order to examine societal impacts and perceptions surrounding STIs, open-ended questions were also posed.

Likert scales were employed in the scoring of the questions. This scale is meant to show how individuals feel about a certain subject. For instance, a 7-point Likert scale used to score agreement would have the following options: strongly disagree, disagree, somewhat disagree, neither agree nor disagree, somewhat agree, and agree. Similar examples can be found for 7-point Likert scales used to measure frequency and satisfaction.

Participants presenting to the walk-in clinic who met the criteria for screening for symptoms of an STI were invited to participate in this study. Recruitment of patients occurred over a period of 2 weeks in May 2021. The researcher was primarily responsible for recruitment, with an enrolled nurse assistant (ENA) working at Du Noon CHC assisting with translation when required. This was arranged and agreed upon by the Du Noon CHC Out Patient Department (OPD) Operational Nursing Manager.

Interviews were conducted in the participant’s language of preference, such as IsiXhosa and Shona, with the help of the ENA, who also did the translation. The interviews were conducted at the Du Noon CHC in a dedicated and private consultation room, located some distance from the general patient flow.

### Data analysis

**FIGURE 2:**
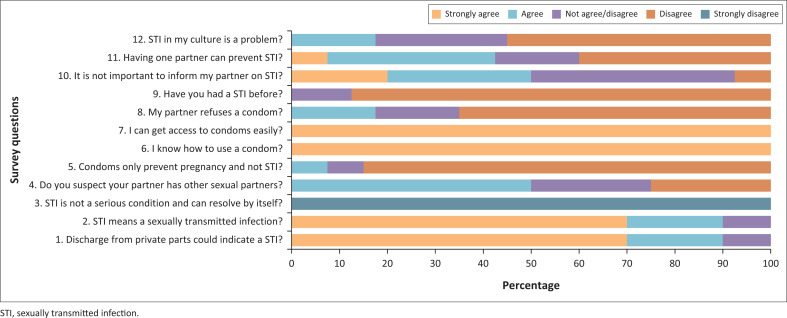
Summary of the survey questions.

All interviews were recorded on audiotape and transcribed verbatim. For the open-ended questions, topics were identified and coded inductively from the text and then grouped together into coherent categories. The answers to the Likert survey questions were examined and explored. Answers are displayed as a summary on Figure 2. When most of the answers were common and towards the ends of the scale, these statements were recorded as important and significant.

The questionnaires and the audio recordings of the interviews were safely kept in a locked cabinet and on a password-protected computer. The lead investigator and their supervisors were the only ones with access to the obtained data.

### Ethical considerations

Ethical clearance to conduct this study was obtained from the University of Cape Town Faculty of Health Sciences Human Research Ethics Committee (No. 068/2021). The Du Noon CHC’s facility manager, the Metro District, and the Provincial Research Committee all gave their approval for the project.

Participants were informed verbally about the study and received a description of the intended use of the data that were collected. The time required for participation and the role of the researcher as non-interfering and non-judgemental were explained. Each participant was asked to sign a consent form by the primary researcher prior to participating in the interviews.

The participants were informed of their freedom to choose what information they disclosed to the researcher and whether or not it may be used. The participants were free to leave at any moment and without incurring any fees. On the day of the interview or in the future, their treatment plan was unaffected by their decision to participate in or withdraw from the study. Participants received assurances that the data collected about them would be kept private. Participants’ names would not be used, either during the recording of the data collected or on the questionnaire.

Owing to the sensitive nature of the issue and the format of in-depth interviews, there was a chance that participants would experience emotional pain or stigma. The researcher carefully searched for indications of discomfort because she was aware of this potential. If this happened during the interview, it was interrupted so that the participant’s needs could be met. There was enough time allotted to convey any meaningful feeling. If a subject showed signs of severe emotional distress, a suitable mental health screening was conducted. After that, a recommendation was made to the most suitable clinician, therapist, or social worker on staff at the CHC.

## Results

The total number of respondents approached by the researcher was 50, and 40 were found to be eligible. Ten respondents were not eligible because of various reasons, such as being younger than 18 years old, or not living in the Du Noon community. Information saturation was obtained from the 40 participants for the open-ended questions. Table 1 shows the gender distribution and whether they were South African or not, and Table 2 provides the age distribution of the participants.

**TABLE 1:** Gender distribution and nationality.

Gender	Male	%	Female	%	Total	%
South African	15	37.5	12	30	27	67.5
Non-South African	9	22.5	4	10	13	32.5

**Total participants**	**24**	**60.0**	**16**	**40**	**40**	**100.0**

**TABLE 2:** Age distribution of the participants.

Age group (years)	Male	%	Female	%
19–25	7	17.5	5	12.5
26–35	12	30	8	20
> 35	5	12.5	3	7.5

According to Figure 2, 90% of participants are generally aware of what an STI is and that a vaginal discharge may be a sign of one (questions 1 and 2). Everyone disagreed on the third question, indicating that they all believed STIs to be significant illnesses that needed to be treated. Many suspected that their partner may have other sexual partners (50%), and some seemed uncertain (25%). The majority of participants knew that condoms may prevent STIs, according to their answers to question 5. Everybody had access to condoms and knew how to use them (questions 6 and 7). It is interesting to note that 20% of respondents were unsure and less than 20% stated their partner refused to use condoms (question 8). In response to question 9, the vast majority (90%) stated that they had never had an STI. We received conflicting answers to our questions (questions 10 and 11) regarding the significance of alerting partners and just having one sexual partner. Responses pertaining to STI and culture also elicited a varied reaction.

The themes that emerged from the open-ended questions were somewhat comparable to those found in the questionnaire; they included the patients’ awareness of STIs, the cultural and religious perspectives on sexual conduct, the connection between socioeconomic status and sexual behaviour, and the sexual behaviour of young individuals. The open-ended questions revealed the following extra themes:

### Sources of knowledge about sexually transmitted infections

The majority of respondents primarily learned about STIs via acquaintances or older siblings. High school life orientation classes simply skimmed the surface when it came to STIs. The primary cause of the participants’ lack of knowledge regarding STI at school was identified as the teachers’ ‘shyness’ while discussing the subject with them.

### Condom usage

When it came to long-term relationships, the majority of participants never used condoms. One explanation for this was that condom use reduces feeling and pleasure, and discourages trust in a partnership. Consuming alcohol appeared to lower the use of condoms:

‘I was head over heels in love with him. Whatever he said goes. But honestly, while I was with him, getting a sexual infection at that time never came to my mind.’ (Participant 1, female, 26 years old)

‘Only the first time – I used a condom, but then afterwards it was direct contact. He says he enjoys being with me like that. I am fine with it because I love him and will do anything to keep him happy.’ (Participant 10, female, 27 years old)

‘I think being in a stable relationship makes it a normal practice to not use condoms. When you are having a fling sex with a friend. You know the thing about “friends with benefits”, since you’re not really in a relationship with that person, and then you use it mostly because you don’t want a bun in the oven.’ (Participant 11, female, 22 years old)

‘If I am drunk at a party or club and then meet a girl, obviously, we are going to have sex without condoms. That’s because sex under the influence of alcohol is always another story. When booze hits hard, you don’t think of condoms, and the girls always love it like that.’ (Participant 7, male, 20 years old)

### Culture and peer pressure

Males were more likely than females to claim that having several sexual partners is encouraged by peer pressure. They also talked about the masculinity of isiXhosa males and how, according to the community and social media, their ethnicity is associated with ideas of superior sexual ability. Many participants have brought up the fact that teens may feel pressured by their peers to start dating while still in high school.

A lack of fulfilment of sexual needs by one sexual partner, a desire for ‘variety,’ ‘lust,’ and the fact that it is a ‘biological thing’ were frequently cited as motivators, particularly among males:

‘Let me explain to you, for example, when a guy and a woman have been together for a long time and she’s busy or tired sometimes, this results in a loss of interest in intimacy, so eventually the guy will have to satisfy himself elsewhere. But there is still love between them, so it can happen that she ends up getting STIs that way.’ (Participant 2, male, 34 years old)

‘Being young, it doesn’t cross your mind of getting STIs by swapping partners. It happened mostly due to peer pressure. I can recall such situations in my younger days where it was just me and my group of friends. Well, at that time it was, what you want, you go for it.’ (Participant 5, male, 22 years old)

‘To be promiscuous it’s something encouraged amongst young IsiXhosa men by older men in our culture, so I think that might be a reason.’ (Participant 7, male, 20 years old)

‘Well, the need for a change, I would say, that’s why, when you meet a new partner, the sex always tastes different. The reaction always surprises you. Usually, you get bored of a monotonous sex life, always same position or same reaction. It’s like the menu is always the same; there is no change. I want to feel different, else the fun in sex is lost. You need to spice up your sex life. But then you never think of STIs at this moment.’ (Participant 17, male, 33 years old)

‘Yes, but it’s not only a man thing; its equality of gender in everything nowadays! I know personally women who got fed up of their guys. This usually happens when the guy is a long-distance driver, so he is away for 3–6 months, and obviously the woman will look around to have sex. She needs to fulfil her natural need. I think you cannot blame her. Her guy is there, but away too long. I can assure you that her guy is also getting his share there wherever he is; he has his needs, and she knows that also.’ (Participant 21, male, 29 years old)

### Socio economic

It was noted that sex was occasionally utilised to obtain money by both men and women who had previously become pregnant. A few individuals connected having sex with having money, meaning that it was a means of escaping poverty:

‘It became a more casual kind of thing when I recall it, and we usually enjoy the sex without the use of condoms. He knows I am on the pill, and the best part is he will usually leave me some extra cash.’ (Participant 10, female, 30 years old)

‘I prefer going out and having sex with more mature guys; I mean working men. They will always buy or bring me something, like a phone or perfume. Take me for a nice meal in a restaurant. I get to enjoy things that are out of my reach. You feel upgraded in status.’ (Participant 28, female, 24 years old)

### Substances

Alcohol consumption easily leads to a state of promiscuous behaviour and makes the intoxicated act with audacity and without restraint, which potentially leads to greater risk taking. The participants stated that the failure to use condoms during intercourse is mostly because of the loss of inhibition caused by alcoholic drinks.

### Social media

Some interviewees stated that social media played a key role in shaping, preserving, and promoting cultural notions of masculinity, gender norms, and sexuality, which, in turn, has an impact on the perspective of the younger generation. Dating sites are utilised to help both men and women find various partners effortlessly. Changing standards and attitudes towards sex are also thought to enhance the risk of STIs through the ease of meeting sexual partners online.

### Partner notification

The likelihood of male individuals being in numerous relationships concurrently was higher than that of female participants. Notification of all partners is challenging because there are few, if any, contact details available. Notifying the other partners was impeded by reluctance to see the partner again and beliefs that they were the source of the virus. Every participant concurred that in order to stop the transmission of STIs, it was critical to get tested for HIV and inform one’s partner. The female participants acknowledged that notification could prevent re-infection and expressed concerns about their overall health or knowledge of their partners’ past relationships:

‘My visit to the clinic today is mostly because I found that my partner has stepped out. He told me he still enjoys sex with me as well. The last time he met with the other guy. He told me that he has been using the condoms, but I know it is a lie since, with me, he never likes to use them. So, I just came for a check-up as I am afraid of catching something down there.’ (Participant 30, female, 36 years old)

‘He wanted out of the blue to try different things while having sex with me. When I asked him, he agreed and stepped out. He says that he feels he enjoys it more with me if we try new things. I am somewhat scared and came for a check-up.’ (Participant 30, female, 36 years old)

‘It’s useless to inform her because I won’t see her again.’ (Participant 17, male, 33 years old)

‘I am a long-distance truck driver. I am a lot of time away from my wife. I am 45 years old. I cannot risk having my marriage blown up by informing my wife of my one-night affairs. I will just get treated, and then after a week I will drive back home to her.’ (Participant 21, male, 29 years old)

‘I have a new girlfriend now, and every now and then I shack up with my ex-girlfriend. If I end up catching something with my ex, now I have to notify my present girlfriend, which will just ruin this new relationship.’ (Participant 35, male, 28 years old)

## Discussion

The aim of the study was to explore factors that contribute to the high incidence of STIs within the Dunoon community. The survey questions indicated that participants knew what an STI was, how it presents, and that treatment is required. They knew that condoms can prevent STIs, how to use them, and had easy access to them. Despite knowledge of STIs and how to prevent them, high-risk behaviour still ensued. Cultural beliefs, having multiple partners, a lack of partner notification, alcohol consumption, and a lack of condom usage are all contributing to the high incidence of STIs.

The open-ended questions delved deeper into these themes. Older men’s cultural conceptions and understandings of sexual interactions appear to have an impact on younger generations. This appears to be fostering a norm and desire for several sexual partners, along with peer pressure and social media. It is also possible that some men’s lack of sexual fulfilment is a sign that they are not in committed, loving relationships. Alternative information regarding the advantages of aiming for a stable, loving relationship is scarce. Teachers seem reluctant to educate students about STIs in a way that is suitable and comprehensive. The socio-economic challenges of the community negatively affect young females. Young females in need of financial support are drawn to males who may be financially empowered and in need of sexual gratification and masculine status. These core values and social problems make it easier to understand why condom usage is low and partner notification is difficult. Adding to the above problem is the high incidence of alcohol usage and the lack of inhibition that results from its use. These findings reiterate those of other studies that showed that high usage of alcohol across South Africa exacerbates risk taking behaviour and interpersonal violence.^20,26,27^

Our study emphasises the significance of raising STI awareness and incorporating sexual and reproductive health into education systems. This was also demonstrated in a 2016 study done in the Platfontein San Community.^8^ Our study highlighted the effects of social economic burden and its relationship to STI, similar to the study done in the Mopani district.^28^ This was also evident in a 2017 study, which explored the link between socioeconomic status and HIV as the most common STI, with the incidence rising in the Free State and Western Cape provinces of South Africa.^29^

Our findings confirmed themes and more general concepts found in earlier research.^4,8,18^ It is a reflection of the other recognised cultural and socioeconomic issues that South African rural communities face.^30^ Language preferences and sexual communication among isiXhosa speakers in Cape Town, South Africa, were examined in a 2011 study published in Health Communication Research. This study examines communication and culture and confirms findings about cultural issues contributing to high rates of STIs.^31^

### Strengths and limitations

Our study added to knowledge in that it explored the cultural influence on sexual behaviour and further highlighted social and economic reasons why women may show risky behaviour despite a good understanding of STIs and prevention.

The results obtained in this study only represented those who attended the Du Noon CHC within those two weeks of the survey (*n* = 40). The results may therefore not adequately reflect the views of the broader community. The survey was done during the COVID-19 level 3 alert lockdown, which may have added extra social and economic strain on participants and changed health-seeking behaviour. Some participants were not fluent in English, and a translator was used. In these interviews, some information may have been lost in translation. Only one interviewer was used because of resource constraints. More males were interviewed than females, possibly skewing the data.

The following provisions were made in this study to promote credibility. The open-ended questions were used to rule out possible biases in the survey. The respondents were free to express their opinions about the questions asked. Maintaining good rapport and setting participants at ease during the interview process. Using verbal and non-verbal cues to encourage confidentiality and a non-judgemental atmosphere. Confidentiality and voluntary inclusion in the study were ensured and reiterated at various points during the interview process. Survey questions had a mixture of positive and negative framing to avoid bias.

By giving each participant the opportunity to complete the survey and by double-checking and examining their responses to the open-ended questions, the triangulation of information was further achieved. To ensure that the recorded information was accurate, participants’ responses were reviewed and read aloud to them throughout the interview process.

### Implications for clinical practice

The most complicated and challenging changes to implement are still behavioural ones. Primary prevention may necessitate multidisciplinary approaches, but clinic-level secondary prevention and treatment strategies need to be strong and current. One area to concentrate on would be youth-friendly services that offer substitute explanations from information gleaned from peer pressure, social media, and culture. Youth empowerment can also be facilitated by youth role models. In addition, community-focussed primary care might employ a greater number of clinical nurses, medical officers, and health promoters who work to improve community health and disseminate messages on social media that could lower risky sexual behaviour.

It has been determined that interventions like working with local communities, changing school policies, and involving parents are helpful in promoting sexual health.^32^ Although these findings may only be applicable to this community, they may have an impact on other communities with comparable populations. Overall, the widespread understanding and framing of HIV as an STI and how it is transmitted may require further investigation. This study filled a gap in the local literature by highlighting how health education challenges, interpersonal relationships, and socioeconomic barriers are still important factors in STI transmission.

There is an urgent need to shift cultural ideology and norms among the youth of the Du Noon community. Adequate and correct information is essential for youth before they begin sexual activity. Healthcare professionals from the CHC could be allowed to give talks in schools. Screening teenagers for sexual and reproductive health is a recommended part of annual preventive health assessments.^33^ This will also be an important step in disseminating STI knowledge among this community’s youth. Regular STI screening and treatment at the community level may aid in the reduction of STI spread.

## Conclusion

Participants had a working understanding of what an STI is, how it manifests, and how to treat it. They had easy access to condoms, knew how to use them, and were aware that they can prevent STIs. High-risk conduct continued despite knowledge of STIs and how to prevent them. The high frequency of STIs is largely because of cultural attitudes, multiple partners, the lack of partner notification, alcohol consumption, and non-use of condoms. The culture, perspectives and understanding of sexual interactions as propagated by older men especially, seem to have had an impact on younger generations. Fortunately, there exists an opportunity here to engender a paradigm shift among the youth in terms of social norms and conventions, which may lead to safer sexual practices and health propagating behaviour. This study brought to light how STI transmission is still largely influenced by interpersonal relationships, socioeconomic hurdles, and problems with health education.
